# Supplementary material for: Nanoscale interface confinement of ultrafast spin transfer torque driving non-uniform spin dynamics
Source: Nat Commun. 2017 Apr 13;8:15007. doi: 10.1038/ncomms15007 (PMC5399283; doi:10.1038/ncomms15007)
Supplement: Supplementary Information — Supplementary Figures, Supplementary Notes and Supplementary References. [file ncomms15007-s1.pdf]

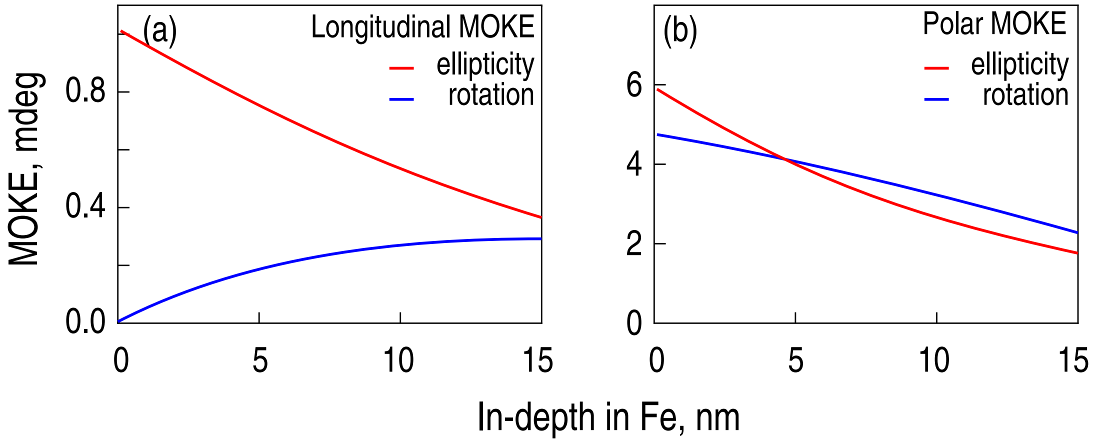

Supplementary Figure 1. MOKE sensitivity. Sensitivity $s(z)$ of (a) longitudinal and (b) polar MOKE rotation (blue lines) and ellipticity (red lines) in a 15 nm-thick Fe film for the p-polarized light.

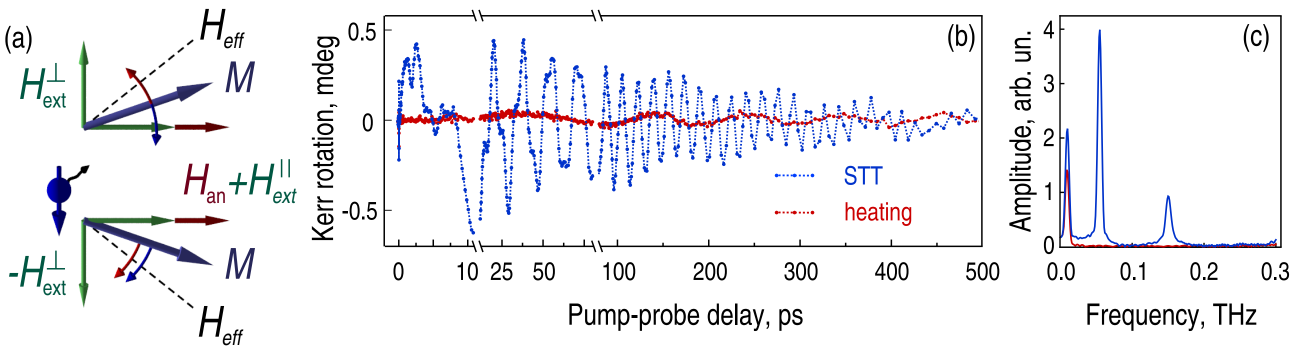

Supplementary Figure 2. Excitation mechanisms. (a) Schematic of the heating and STT-induced magnetization dynamics for two opposite directions of $H_{\text{ext}}^{\perp}$, a projection of the external magnetic field perpendicular to the magnetization M of the collector. Red and blue short arrows depict the initial dynamics of the magnetization induced by the heating and STT mechanisms, respectively. (b-c) Heating- (red) and STT (blue)-induced contributions to the polar time-resolved MOKE signal (b) and their spectra (c).

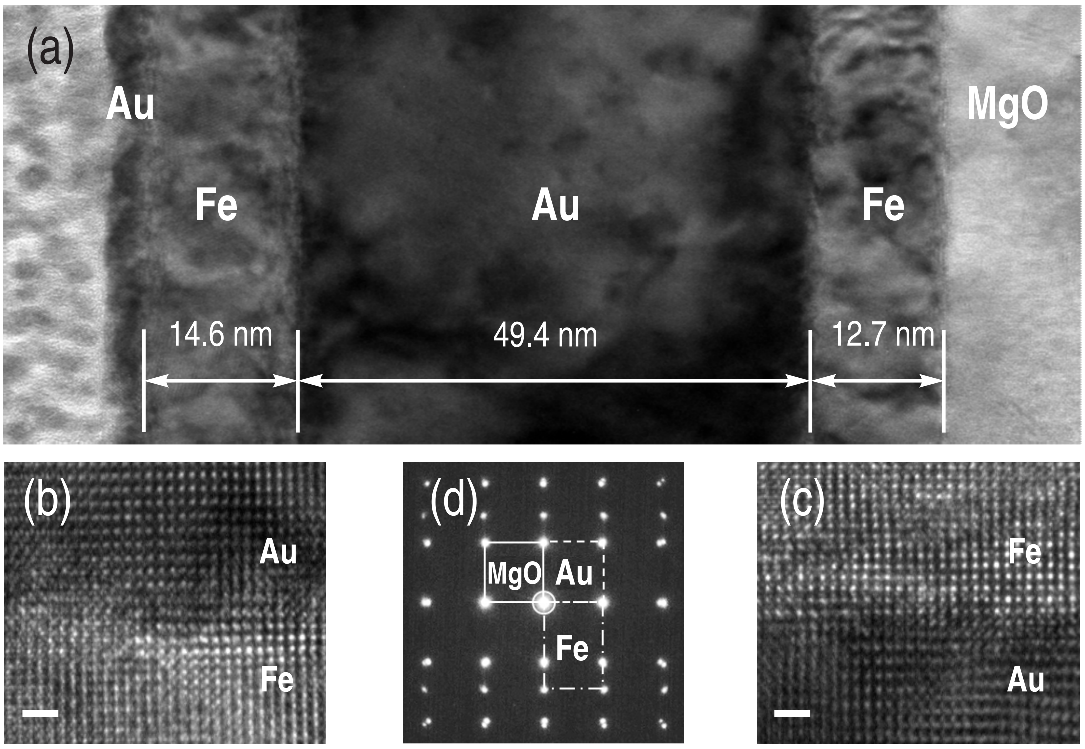

Supplementary Figure 3. Electron microscopy images. (a) Au/Fe/Au/Fe/MgO(001) cross-section transmission electron microscopy (TEM) image. (b-c) High-resolution TEM images of flat Fe/Au and Au/Fe interfaces. The horizontal bar is 1 nm long. (d) Electron diffraction image.

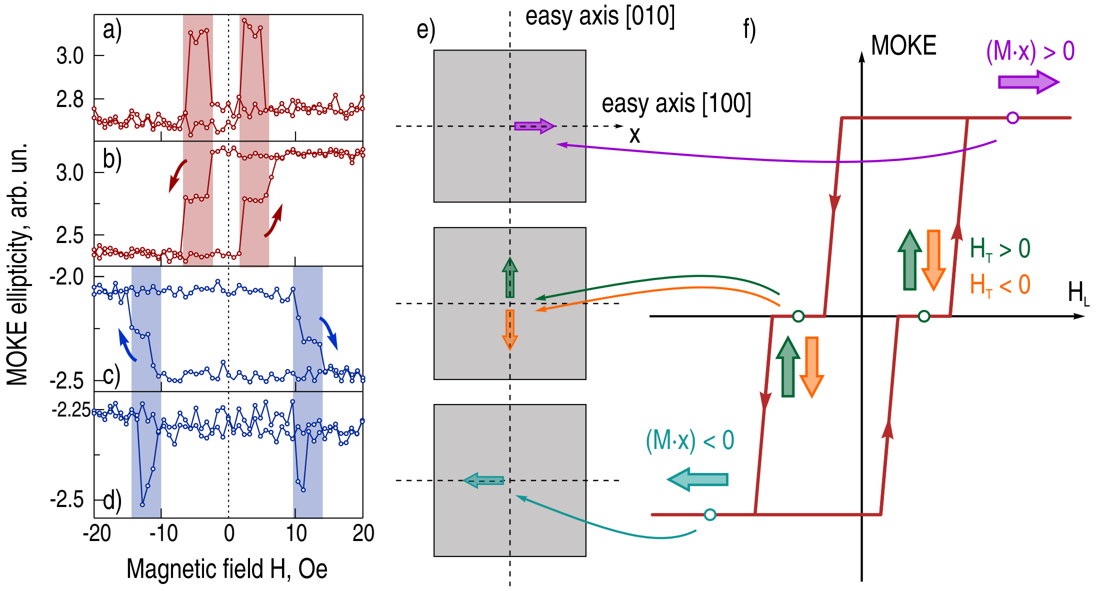

Supplementary Figure 4. Switching between magnetic states. (a-d) Hysteresis loops measured with MOKE from the emitter (a-b) and the collector (c-d) Fe films. In (a,d) H_T is swept whereas H_L is kept constant, in (b,c) the situation is reversed. (e-f) Schematic of the magnetization switching using main H_L and auxiliary H_T magnetic fields applied perpendicular to each other along the two easy axes of magnetic anisotropy in Fe(100). The four coloured points at the sketch of the MOKE hysteresis loop (f) correspond to the four possible directions of magnetization (e).

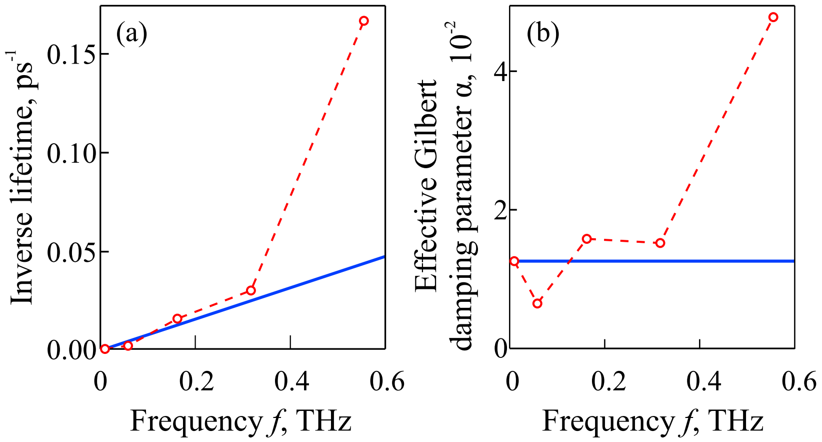

Supplementary Figure 5. Damping of the magnetization precession. (a) Inverse lifetime of the spin wave eigenmodes and (b) effective Gilbert damping parameter α (both open circles) as obtained from fitting the decay time in the oscillatory data and from calculating the corresponding α values (see text above). The solid blue line illustrates the expected lifetimes if the effective damping parameter of the 1-st and higher modes were the same as for the uniform precession eigenmode with $k = 0$.

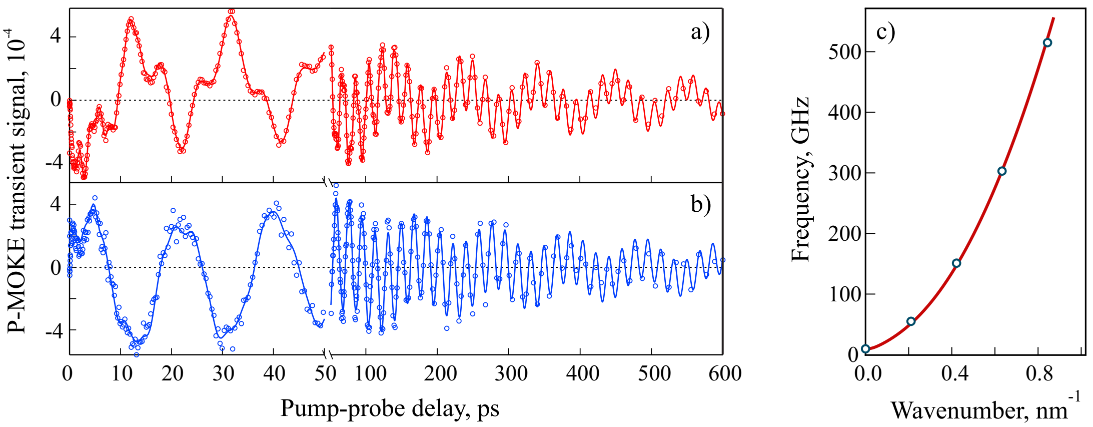

Supplementary Figure 6. Time-resolved MOKE signals. Transient P-MOKE rotation (a) and ellipticity (b) signals obtained on another sample with a different thickness of the collector (13.2 nm instead of 12.7 nm, according to the scanning electron microscopy). Solid lines are the results of the fit with a set of five decaying oscillatory modes. (c) Frequencies from the fit of the P-MOKE data (open circles) and dispersion of the spin wave eigenmodes (solid line) with the Fe magnon stiffness of 280 meV A^2.

Supplementary Note 1

Separation of the polar and longitudinal MOKE contributions

The spin transfer torque (STT)-induced contribution to the magnetization dynamics is given by [1]:

$$\frac{1}{\gamma}\frac{\partial \mathbf{M}}{\partial t}\bigg|_{\mathrm{STT}} = \lambda \mathbf{M} \times [\boldsymbol{\mu}(t) \times \mathbf{M}]. \tag{1}$$

Here λ is the scaling factor, $\boldsymbol{\mu}$ is the magnetic moment carried by the spin current and $\mathbf{M}$ is the magnetization of a ferromagnet. It is seen that the STT-induced magnetization dynamics has different parity with respect to both $\boldsymbol{\mu}$ and $\mathbf{M}$. Whereas variations of $\mathbf{M}$ are odd with respect to $\boldsymbol{\mu}$, at the same time they are even with respect to $\mathbf{M}$ itself. In other words, consider the collector magnetized transversely along the y-axis ($M_y \neq 0$), and the emitter magnetization (proportional to $\boldsymbol{\mu}$) along the x-axis (longitudinal). Here, upon the impulsive STT excitation the collector magnetization $\mathbf{M}$ acquires a x-projection M_x. The sign of M_x replicates the sign of μ_x and is independent of the sign of M_y.

Further, the magnetization precession around its equilibrium (parallel to the y-axis) leads to the emergence of the dynamic polar (out-of-plane) component M_z. Moreover, at the initial stage of the precession $\Delta M_z \propto dM_z/dt \propto H_{\mathrm{an}} M_x$, where H_{an} is the anisotropy field parallel to the y-axis and thus the non-perturbed direction of the magnetization M_y. It is seen that the sign of the polar component M_z is determined by both signs of the projections μ_x, M_y, whereas the sign of the longitudinal component M_x depends on the sign of the μ_x only. Owing to that, it becomes possible to separate the polar MOKE contribution from the longitudinal one when dealing with four datasets measured in various configurations of the equilibrium magnetizations, $\pm\mu_x$, $\pm M_y$ (Figure 2c-d in the main Manuscript). Indeed, consider the notation where U, D (R, L) as in up, down (right, left) denote the magnetization and magnetic moment directions parallel and antiparallel to the y (x) axis. The experimental configurations with two orthogonal magnetizations discussed above can be written as RU, RD, LU, LD, where the first and the second symbols represent the magnetization of the emitter (proportional to μ) and the collector M, respectively. As such, odd with respect to μ, the pure longitudinal dynamics of Kerr rotation is given by

$$\varphi_L = \frac{1}{4}(\varphi_{\mathrm{RU}} + \varphi_{\mathrm{RD}} - \varphi_{\mathrm{LU}} - \varphi_{\mathrm{LD}}). \tag{2}$$

Further, odd (even) with respect to the magnetization of the emitter (collector), the polar Kerr dynamics is given by

$$\varphi_P = \frac{1}{4}(\varphi_{\mathrm{RU}} - \varphi_{\mathrm{RD}} + \varphi_{\mathrm{LU}} - \varphi_{\mathrm{LD}}). \tag{3}$$

Supplementary Note 2

MOKE in-depth sensitivity

Despite having no total magnetic moment, standing spin waves can be visualized in MOKE experiments because of the in-depth selective sensitivity of MOKE. Supplementary Figure 1 illustrates the calculated specific MOKE rotation and ellipticity for the 15 nm-thick Fe films. The data were calculated using the layer-by-layer approach based on the medium boundary and propagation matrices [2–5] for the p-polarized incident beam. The total MOKE response θ, ε is given by a convolution of the sensitivity $s(z)$ and the certain in-depth profile of magnetization $m(z)$:

$$\theta, \varepsilon = \int_0^d s_{\theta,\varepsilon}(z) m(z) dz \tag{4}$$

Two main considerations can be inferred from the data shown in Supplementary Figure 1. Firstly, it is seen that longitudinal MOKE rotation and ellipticity are predominantly sensitive to the opposite interfaces of the film. On the contrary, polar MOKE rotation and ellipticity exhibit very similar sensitivities to local magnetization $m(z)$. As such, the differences in the transient rotation and ellipticity in the case of the longitudinal MOKE could be attributed to a different dynamics at the two interfaces of the Fe film. Similarly, in-depth MOKE sensitivity is responsible for the detected amplitudes of the excited spin wave eigenmodes in MOKE signals. We also note that the oscillating transient magnetic moment in the Au spacer might also contribute to the MOKE signals, thus making the analysis of the amplitudes non-trivial. As such, it is difficult to use MOKE amplitudes for drawing conclusions on the STT spatial profile Furthermore, considering a simple exponentially decaying shape for the interface STT contribution can be misleading as the detected amplitudes of the spin wave eigenmodes might be enhanced or suppressed by the resonances in the excitation of particular modes. In fact, this resonant behaviour is supported by the analysis of the lifetimes (and effective damping parameter) of the spin wave engenmodes (see Supplementary Note 6). It is seen there that the 4-th eigenmode is very different to the other ones up to the 3-rd, having a much stronger effective damping than can be expected.

Supplementary Note 3

Separation of the heating and STT contributions in the spin waves excitation

The experiments described in the main Manuscript were performed without external magnetic field. In order to study the heating effect on the transport-induced magnetization dynamics in Fe/Au/Fe trilayers, we performed additional measurements with an external magnetic field $\vec{H}$ on. This magnetic field was applied in the plane of the sample at an angle with respect to the easy axes of the two Fe films, so that the magnetizations of the emitter and the collector were aligned orthogonally to each other (see Supplementary Note 5). In this configuration (Supplementary Figure 2,a), the equilibrium direction of the collector magnetization M is determined by an interplay of the external magnetic field $\boldsymbol{H}_{\text{ext}} = \boldsymbol{H}_{\text{ext}}^{\parallel} + \boldsymbol{H}_{\text{ext}}^{\perp}$ and the anisotropy field $\boldsymbol{H}_{\text{an}}$. The thermal action of the ultrashort electron pulse results in heating of the collector and thus quenching its magnetic anisotropy H_{an}. As such, the equilibrium direction of the magnetization M shifts giving rise to the precessional dynamics around H_{eff} with the frequency $f_0 \approx 10$ GHz [6].

Supplementary Figure 2,a illustrates this mechanism along with the STT excitation discussed above. Note that according to the Supplementary Equation 1, the STT (heating) mechanism is even (odd) with respect to the external field $\mathbf{H}_{\text{ext}}$. Thus, the direction of the heating-induced precession reverses together with the perpendicular projection of the applied external field $\mathbf{H}_{\text{ext}}^{\perp}$. We note, however, that the STT mechanism does not depend on the external magnetic field as long as the directions of the magnetizations of both emitter and collector are set. Thus, obtaining the MOKE data for the two opposite projections of the external field $\mathbf{H}_{\text{ext}}^{\perp}$ perpendicular to the equilibrium collector magnetization (but keeping the magnetization M in place), we can separate the heating and STT contributions (Supplementary Figure 2,b). Whereas the STT-driven dynamics (blue symbols) has a much larger amplitude and can boast a rich spectrum of frequencies, the heating-induced magnetization dynamics (red symbols) is small and consists of a single FMR mode only (Supplementary Figure 2,b-c). The latter can be explained invoking the good heat conductivity of Fe along with the large duration of heat pulses as compared to spin current ones. Both factors facilitate the excitation of the homogeneous precession with $k = 0$ (FMR) and inhibit any inhomogeneous (with $k > 0$) dynamics of thermal origin.

Supplementary Note 4

Transmission electron microscopy

The Fe/Au/Fe/MgO(001) samples were capped with a 3 nm-thick Au protective layer. The structure of the samples was characterized using a transmission electron microscope (TEM) TITAN 80-300 (FEI, USA) equipped with a corrector of spherical aberration at image side. The microscope was operated at 300 kV. An example of cross-section specimen prepared by means of the Focused Ion Beam lift-out technique is shown in Supplementary Figure 3. The cross-section was prepared at the spot used for the optical measurements. The micro-structural study reveals that both Fe and Au films grow epitaxially and the roughness of Fe/MgO and Fe/Au interface varies from 0.7 to 1.5 nm. The precise measurements of the layers thicknesses yield the values of 12.7 and 14.6 nm for the Fe collector and emitter, respectively, and 49.4 nm for the Au spacer. We note that the collector thickness obtained from the microscopy is somewhat smaller than the one used in the main manuscript for the data fitting. However, this deviation can be mitigated by adjusting the magnon stiffness of Fe. It is known, for example, that thin Fe films of up to 24 monolayers demonstrate significantly lower stiffness (160 meV$\cdot$A^2 as compared to 280 meV$\cdot$A^2 for the bulk Fe) [7]. In our case, the thickness obtained using the microscopy results in $D \approx$ 200 meV$\cdot$A^2, in between of these two values. Moreover, the lattice mismatch at the MgO/Fe interface leads to the appearance of defects in the Fe film, which can also modify the effective magnon stiffness D.

Supplementary Note 5

Realizing orthogonal magnetic configurations

Static magnetic properties of the emitter and collector Fe layers were characterized by employing the magneto-optical Kerr effect (MOKE) measured independently. A thick (55 nm) Au spacer prevented magnetic coupling of the two Fe layers, as well as optical access to the collector from the emitter side and vice versa. As such, we performed simultaneous characterization of the magnetic states of the collector and emitter using MOKE with two laser beams. We note that thin Fe films have two in-plane orthogonal easy axes corresponding to the $\langle 100 \rangle$ and $\langle 010 \rangle$ directions (see also Ref. [8]). In our experiments, the sample was placed in such a way that one of these axes was in the incidence plane. We found that both magnetizations were switched to the opposite direction along one of the easy axes while the magnetic field in the longitudinal MOKE geometry H_L was swept from -20 to 20 Oe.

In order to force the magnetization along the other easy axis we have applied a small auxiliary transverse magnetic field H_T perpendicular to the main one. We found that for the values of H_T as small as 4 Oe the switching of magnetization acquired a step in the middle (Supplementary Figure 4,b-c) corresponding to the zero MOKE rotation and ellipticity. At the same time, when sweeping the transverse field while keeping the small H_L on, we observed a sharp feature with a non-zero the MOKE rotation and ellipticity response (Supplementary Figure 4,a,d). This indicates that the magnetization switching proceeds via an intermediate state which corresponds to the orthogonal orientation of the magnetization, along the second easy axis in the film. As such, when sweeping the main magnetic field H_L across the hysteresis loop in the presence of the auxiliary field H_T which could be positive or negative, we were able to attain any of the four orientations of the magnetization in the Fe film (Supplementary Figure 4,e-f).

It is seen in Supplementary Figure 4,a-d that the hysteresis loops of the emitter and the collector have different widths, *i.e.* the coercive fields of the two films are unequal. This allows for the realization of a orthogonal configuration, where the magnetizations of the emitter and the collector are perpendicular to each other. For instance, if in the magnetic set-up used for the measurements shown in Supplementary Figure 4,a-b longitudinal magnetic field of ≈ 12 Oe was applied, the emitter and the collector were magnetized along the longitudinal and transverse axes, respectively. Varying the roles of the longitudinal and transverse magnetic fields (main or auxiliary) as well as their signs, it was possible to realize all 8 orthogonal magnetic configurations of the multilayer sample.

Supplementary Note 6

Excitation and lifetimes of higher-order spin wave eigenmodes

As it has been argued in the main Manuscript, there are temporal and spatial requirements for the efficient excitation of a spin wave. Here, a spin wave with the frequency f and wavevector k is effectively excited if the stimulus duration is shorter than $1/2f$ and localized in the π/k-wide region in space. Indeed, a perturbation confined to the short λ_{STT} length scale has a broad lineshape in $k-$space with a characteristic width of about the inverse λ_{STT}. Further, Figure 4,c in the main Manuscript illustrates that for the standing spin waves with open ends, the critical STT excitation depth is about a quarter of the wavelength. This means that the eigenmode with k_5 is not efficiently excited if the characteristic STT depth $\lambda_{\mathrm{STT}} > 1/4 \times 2\pi/k_5 \approx 1.5$ nm. We note, however, that there are other possible limitation mechanisms related to the lifetime of the eigenmodes. From the fitting procedure, the eigenmode with $k_4 = 4\pi/d$ and the period $T_4 \approx 2$ ps was found to live for about 6 ps. The eigenmodes with even larger k most likely have even shorter lifetimes (comparable to their periods or smaller) and thus render invisible in our experiments.

However, even if excited, the eigenmode might not be observable in the time-resolved MOKE measurements due to its quick damping. In Supplementary Figure 5,a-b we plot the inverse lifetime of the modes t_i^{-1} and effective Gilbert damping parameter α (open circles) obtained from the fit procedure as a function of the eigenmodes number. Specifically, we fitted the experimental data with a set of five oscillatory functions with frequencies given by Equation 2 in the main Manuscript and their amplitudes and lifetimes as free parameters. Further, we calculated the corresponding effective Gilbert damping $\alpha = (2\pi f_i \tau_i)^{-1}$ for the spin wave eigenmodes (Supplementary Figure 5,b). The blue solid lines indicate the inverse lifetimes τ^{-1} and effective damping α assuming that the latter is equal for all modes. In other words, along the blue line the product $f_i \tau_i = f_0 \tau_0 = \mathrm{const}$, where f_i is the frequency of the i-th eigenmode. It is seen that the lifetimes of the spin wave eigenmodes up to the 3-rd are scattered around the blue line whereas the 4-th eigenmode has a much shorter lifetime (or stronger effective damping) than predicted by the equal damping model. This hints at the possibility of even stronger effective damping for even higher spin wave eigenmodes and could alter the estimation of the spatial localization of the STT perturbation to even lower values, as in this case higher eigenmodes would be excited and immediately damped. The upper estimation limit of the STT length $\lambda_{\mathrm{STT}} \approx 2$ nm remains therefore unchanged.

Supplementary Note 7

The role of thickness of the Fe collector

Here we show the polar MOKE data obtained on a different sample with a slightly thicker collector layer (Supplementary Figure 6). We fitted the data in Supplementary Figure 6,a with a set of five exponentially decaying oscillating functions where their frequencies were free parameters. After that, the obtained frequencies were fitted the Equation 2 in the main Manuscript using the bulk magnon stiffness of Fe $D = 280$ meV·A^2. It is seen that the frequencies present in the time traces (Supplementary Figure 6,a-b) and obtained from the fitting procedure are in an excellent agreement with those given by the spin wave dispersion in Fe (Supplementary Figure 6,c). Note that the considerations discussed in Supplementary Note 4 regarding the possible modification of the Fe magnon stiffness D apply here as well.

Supplementary References

[1] Slonczewski, J. C. Current-driven excitation of magnetic multilayers. J. Magn. Magn. Mater. **159**, L1–L7 (1996).
[2] Zak, J., Moog, E. R., Liu, C. & Bader, S. D. Universal approach to magnetooptics. J. Mag. Mag. Mater. **89**, 107 (1990).
[3] Traeger, G., Wenzel, L. & Hubert, A. Computer Experiments on the Information Depth and the Figure of Merit in Magnetooptics. Phys. stat. sol. (a) **131**, 201–227 (1992).
[4] Hamrle, J. *et al.* Analytical expression of the magneto-optical Kerr effect and Brillouin light scattering intensity arising from dynamic magnetization. J. Phys. D: Appl. Phys. **43**, 325004 (2010).
[5] Wieczorek, J. *et al.* Separation of ultrafast spin currents and spin-flip scattering in Co/Cu(001) driven by femtosecond laser excitation employing the complex magneto-optical Kerr effect. Phys. Rev. B **92**, 174410 (2015).
[6] Carpene, E. *et al.* Ultrafast three-dimensional magnetization precession and magnetic anisotropy of a photoexcited thin film of iron. Phys. Rev. B **81**, 060415(R) (2010).
[7] Prokop, J. *et al.* Magnons in a Ferromagnetic Monolayer Phys. Rev. Lett. **102**, 177206 (2009).
[8] Zhan, Q.-F. *et al.* Magnetic anisotropies of epitaxial Fe/MgO(001) films with varying thickness and grown under different conditions. New. J. Phys. **11**, 063003 (2009).
